# Swimming Turned on Its Head: Stability and Maneuverability of the Shrimpfish (*Aeoliscus punctulatus*)

**DOI:** 10.1093/iob/obz025

**Published:** 2019-10-10

**Authors:** F E Fish, R Holzman

**Affiliations:** 1 Department of Biology, West Chester University, West Chester, PA 19383, USA; 2 School of Zoology, Tel Aviv University and the Inter-University for Marine Sciences in Eliat, Eliat 88103, P.O. Box 469, Israel

## Abstract

The typical orientation of a neutrally buoyant fish is with the venter down and the head pointed anteriorly with a horizontally oriented body. However, various advanced teleosts will reorient the body vertically for feeding, concealment, or prehension. The shrimpfish (*Aeoliscus punctulatus*) maintains a vertical orientation with the head pointed downward. This posture is maintained by use of the beating fins as the position of the center of buoyancy nearly corresponds to the center of mass. The shrimpfish swims with dorsum of the body moving anteriorly. The cross-sections of the body have a fusiform design with a rounded leading edge at the dorsum and tapering trailing edge at the venter. The median fins (dorsal, caudal, anal) are positioned along the venter of the body and are beat or used as a passive rudder to effect movement of the body in concert with active movements of pectoral fins. Burst swimming and turning maneuvers by yawing were recorded at 500 frames/s. The maximum burst speed was 2.3 body lengths/s, but when measured with respect to the body orientation, the maximum speed was 14.1 body depths/s. The maximum turning rate by yawing about the longitudinal axis was 957.5 degrees/s. Such swimming performance is in line with fishes with a typical orientation. Modification of the design of the body and position of the fins allows the shrimpfish to effectively swim in the head-down orientation.

## Synopsis

Schwimmen auf den Kopf gestellt: Stabilität und Wendigkeit des Schnepfenmesserfisches (*Aeoliscus punctulatus*) (Swimming Turned on Its Head: Stability and Maneuverability of the Shrimpfish (*Aeoliscus punctulatus*)) Die typische Ausrichtung eines schwebend schwimmenden Fisches ist mit dem Bauch nach unten und dem Kopf nach vorne entlang der Längsachse zeigend. Verschiedene stärker abgeleitete Teleostei richten den Körper jedoch vertikal aus um zu fressen, sich zu verbergen oder Greifbewegungen durchzuführen. Der gepunktete Schnepfenmesserfisch (*Aeoliscus punctulatus*) hält eine vertikale Ausrichtung aufrecht, wobei der Kopf nach unten zeigt. Diese Haltung wird durch das Schlagen der Flossen aufrechterhalten, da die Position des Formschwerpunkts nahezu dem Gewichtsschwerpunkt entspricht. Der Schnepfenmesserfisch schwimmt mit dem Rücken des Körpers nach vorne. Die Körperquerschnitte sind spindelförmig mit einer abgerundeten Vorderkante am Rücken und einer sich verjüngenden Hinterkante am Bauch. Die Mittelflossen (dorsal, kaudal, anal) sind entlang des Bauchs des Körpers positioniert und werden geschlagen oder als passives Ruder verwendet, um die Bewegung des Körpers in Übereinstimmung mit aktiven Bewegungen der Brustflossen zu bewirken. Explosionsartiges Schwimmen und Wendemanöver durch Gieren wurden mit 500 frames/s aufgezeichnet. Die maximale Geschwindigkeit explosionsartigen Schwimmens betrug 2, 3 Körperlängen/s, aber gemessen in Bezug auf die Körperorientierung betrug die maximale Geschwindigkeit 14, 1 Körpertiefen/s. Die maximale Drehrate durch Gieren um die Längsachse betrug 957, 5 Grad/s. Diese Schwimmleistung entspricht Fischen mit einer typischen Ausrichtung. Durch die Modifikation des Körperdesigns und der Position der Flossen kann der Schnepfenmesserfisch effektiv mit dem Kopf nach unten schwimmen. Translated to German by F Klimm (frederike.klimm@biologie.uni-freiburg.de)

La natación al revés: estabilidad y maniobrabilidad del pez *Aeoliscus punctulatus* (Swimming Turned on Its Head: Stability and Maneuverability of the Shrimpfish (*Aeoliscus punctulatus*)) La orientación típica de un pez neutralmente flotante es con la abertura hacia abajo y la cabeza apuntando hacia delante a lo largo del eje longitudinal. Sin embargo, varios teleósteos avanzados reorientarán el cuerpo verticalmente para comer, ocultarse o agarrarse. El pez *Aeoliscus punctulatus* mantiene una orientación vertical con la cabeza apuntando hacia abajo. Esta postura se mantiene mediante golpes de las aletas mientras la posición del centro de flotabilidad casi corresponde al centro de masa. El pez nada con el dorso del cuerpo en movimiento anterior. Las secciones transversales del cuerpo tienen un diseño fusiforme con un borde delantero redondeado en el dorso y un borde posterior que se estrecha en la abertura. Las aletas medianas (dorsales, caudales, anales) se colocan a lo largo de la abertura del cuerpo y se golpean o se usan como un timón pasivo para efectuar el movimiento del cuerpo en concierto con los movimientos activos de las aletas pectorales. Se registraron maniobras de natación en ráfaga y de vueltas por guiñada a 500 cuadros/s. La velocidad máxima natación en ráfaga fue de 2, 3 longitudes corporales/s, pero cuando se midió con respecto a la orientación corporal, la velocidad máxima fue de 14, 1 profundidades corporales/s. La velocidad máxima de vuelta por desviarse alrededor del eje longitudinal fue de 957, 5 grados/s. Tal rendimiento de natación está en línea con los peces con una orientación típica. La modificación del diseño del cuerpo y la posición de las aletas permite que el pez *Aeoliscus punctulatus* nade efectivamente en la orientación de cabeza hacia abajo. Translated to Spanish by YE Jimenez (yordano_jimenez@brown.edu)

A natação de cabeça para baixo: Estabilidade e manobrabilidade do peixe-camarão (*Aeoliscus punctulatus*) (Swimming Turned on Its Head: Stability and Maneuverability of the Shrimpfish (*Aeoliscus punctulatus*)) A orientação típica de um peixe de flutuação neutra é com o ventre para baixo e a cabeça apontada anteriormente ao longo do eixo de oscilação longitudinal. No entanto, vários teleósteos derivados reorientam o corpo verticalmente para alimentação, ocultação ou prensão. O peixe-camarão (*Aeoliscus punctulatus*) mantém uma orientação vertical com a cabeça apontada para baixo. Essa postura é mantida pelo batimento das nadadeiras, já que a posição do centro de empuxo quase corresponde ao centro de massa. O peixe-camarão nada com o dorso do corpo movendo-se anteriormente. As seções transversais do corpo têm um desenho fusiforme com uma borda de ataque arredondada no dorso e uma de fuga no ventre. As nadadeiras medianas (dorsal, caudal e anal) são posicionadas ao longo do ventre e são batidas ou usadas como um leme passivo para efetuar o movimento do corpo em conjunto com os movimentos batidos das nadadeiras peitorais. As manobras explosivas de natação e de guinada foram registradas a 500 quadros por segundo. A velocidade máxima de explosão foi de 2, 3 comprimentos de corpo por segundo, mas quando medida em relação à orientação do corpo, a velocidade máxima foi de 14, 1 vezes a profundidade do corpo por segundo. A taxa máxima de rotação através da guinada em torno do eixo longitudinal foi de 957, 5 graus por segundo. Esse desempenho de natação está de acordo com peixes com uma orientação típica. A modificação do desenho do corpo e a posição das barbatanas permitem que os peixes-camarão nadem efetivamente de cabeça para baixo. Translated to Portuguese by G Sobral (gabisobral@gmail.com)

## Introduction

How fishes orient their bodies in the aquatic environment is an important parameter related to swimming performance and their biological role. Typical orientation is in the prone position with the longitudinal axis of the body parallel to the earth-based horizontal plane (Webb 2006). When the fore and aft of a free-floating body are at the same level so that the gravitational and buoyancy forces are balanced, such an orientation is said to be in trim, which maintains longitudinal stability (Burcher and Rydill 1994; Renilson 2015). In trim, the longitudinal axis of a body would parallel the horizontal plane. The longitudinal stability is affected hydrostatically by the relative distance of the center of mass (CM) with respect to the center of buoyancy (CB) (Weihs 1973, 2002). The most stable orientation is affected by the positions of these centers with the CB directly above the CM (Weihs 1973, 1993, 2002; Borelli 1989; Videler 1993; Eidietis et al. 2002). The up-thrust positioned at the CB counters the downward force due to gravity from the CM. This arrangement generates correcting torques to counter instabilities and provide longitudinal stability against pitching moments (i.e., up and down rotation about a transverse axis), and provide transverse stability to resist rolling moments (i.e., rotation around the longitudinal axis) due to internal or external perturbations (Renilson 2015). The greater the vertical distance between the CM and the CB, the greater the resistance to pitching and rolling. Conversely, a large displacement between the CM and the CB reduces the ability to maneuver. To enhance turning performance, particularly with regard to pitch and roll, fishes maintain trim hydrodynamically (i.e, fluids in motion) as well as hydrostatically (i.e., fluids at rest in static equilibrium). Hydrodynamic trim control (i.e., dynamic stability) is affected by the use of fixed (e.g., head and body shape, keels) and mobile (e.g., pectoral and pelvic fins) control surfaces (Breder 1926; Harris 1936, 1938; Alexander 1990; Fish and Shannahan 2000; Fish and Lauder 2006, 2017). Mobile control surfaces generate hydrodynamic lift forces to stabilize the body and maintain trim but can vector the lift generated to maneuver and change body trajectory.

Prone orientation for fishes has advantages with regard to swimming performance. The elongated body along the longitudinal axis enhances streamlining for faster locomotion. The body is shaped in a fusiform design that effectively reduces the pressure component of drag (Webb 1975). Elongation of the body permits bending for small-radius, yawing turns. Elongation along the longitudinal axis also provides the caudal fin with a long lever arm (i.e., distance from the CM to fin) to generate large torques like a rudder and maintain directional stability or promote rapid yawing turns. Displacement of mass toward the head provides an inertial mass that limits recoil in the anterior end from the reciprocating propulsive tail movements and thereby reduces the energetic cost of swimming (Lighthill 1971, Webb et al. 1984; Webb 1992).

Despite the advantages of maintaining horizontal trim for typical fishes, various fishes can swim or float with different orientations. The upside-down catfish (*Synodontis nigriventris*) swims with the venter of the body directed upward (Norman and Greenwood 1975; Meyer et al. 1976; Blake and Chan 2007). The seahorse (genus *Hippocampus*) swims with its body in a vertical orientation as the head is canted horizontally or downward. This orientation allows the sea horse to use its prehensile tail to wrap around vegetation but limits the speed and swimming performance (Wood 1976). The dwarf sea horse (*Hippocampus zosterae*) is considered to be the slowest swimming fish (Carwardine 2013). The elongate oarfish (*Regalecus glesne*) was observed to swim in the open ocean in a head-up vertical orientation (Benfield et al. 2013). The trumpetfish (genus *Aulostomus*, family Aulostomidae, suborder Syngnathoidei) uses a head-down vertical orientation when hunting (Aronson 1983; Helfman and Winkelman 2010; Betancur-R et al. 2017). In addition, the trumpetfish will vertically align itself with objects in its environment (e.g., gorgonians, sponges, ropes) for concealment from prey (Scarr 1980; Aronson 1983).

**Fig. 1:**
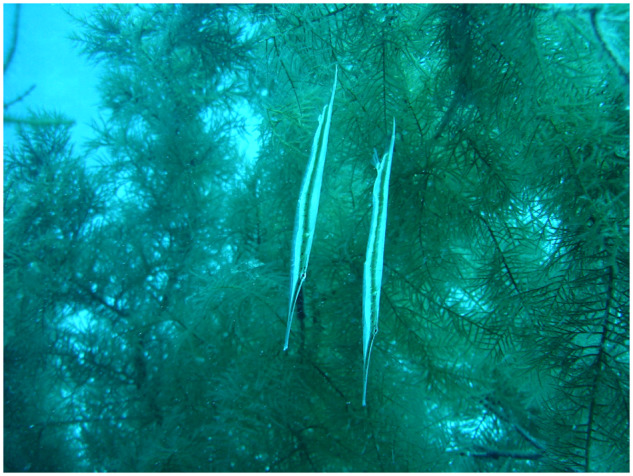
Shrimpfishes in their natural environment in the Gulf of Aqaba orienting vertically to soft coral.

Also within the Syngnathoidei, although not considered a sister clade with trumpetfishes, are the shrimpfishes (also called “razorfish”) in the family Centriscidae that swim in a head-down vertical orientation (Atz 1962; Klausewitz 1963, 1966; Marshall 1966; Norman and Greenwood 1975; Consi et al. 2015; Betancur-R et al. 2017). Unlike most other fishes, shrimpfishes orient the body with a head-down orientation. This orientation is believed to be associated with protection or camouflage in their natural environment. Shrimpfishes use a head-down vertical orientation to hide among the long needle-like spines of sea urchins (e.g., *Diadema antillarum*) (Randall et al. 1964; Marshall 1966; Norman and Greenwood 1975; Helfman et al. 1997). While on SCUBA, the authors were able to observe several shrimpfishes at a submerged depth of 30 m in the Gulf of Aqaba. The fishes maintained a head-down orientation as they hid among the branches of a soft coral (personal observation; Fig. 1).

**Fig. 2:**
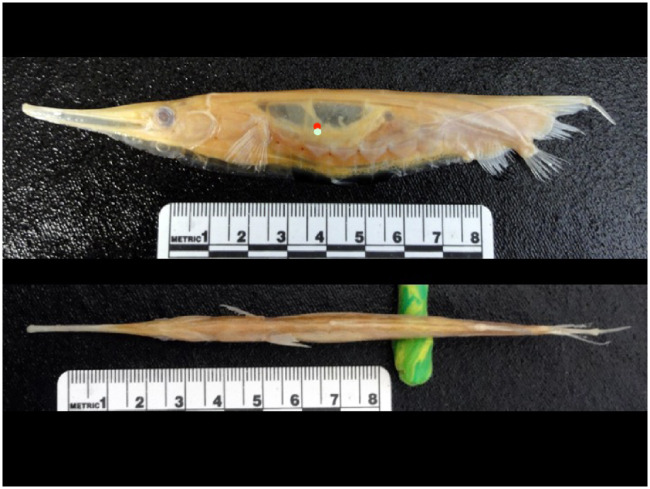
Lateral (top) and ventral view of a shrimpfish (*A. punctulatus*) showing highly compressed body and position of the translucent swim bladder. The positions of the CM (red circle) and the CB (white circle) are indicated in the lateral view of a shrimpfish.

**Fig. 3:**
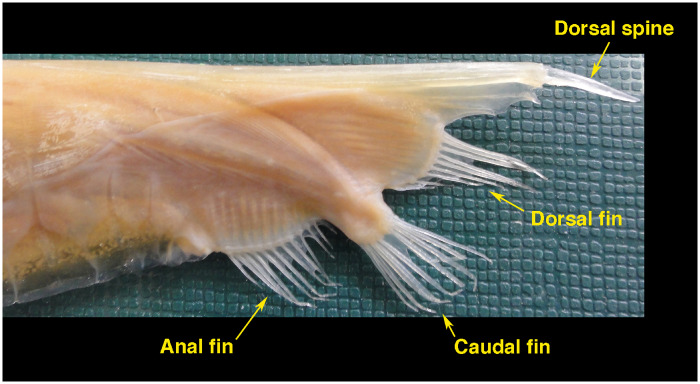
Posterior of shrimpfish showing modification of the position of the dorsal, caudal, and anal fins.

Shrimpfishes are in the family Centriscidae with two genera (*Aeoliscus*, *Centriscus*) and four extant species (Bond 1979). Shrimpfishes are relatively old as fossils of *Aeoliscus* date back to the Oligocene–Miocene of Europe (Parin and Micklich 1996). The shrimpfish has an elongate, strongly compressed body (Fig. 2) that is encased in a “straight-jacket” of translucent bony plates (Marshall 1966). The swim bladder is readily observable through the body armor. The rigid body has a shape that was described as an “edible pea pod” with a rounded dorsal edge and a ventral knife-like keel (Norman and Greenwood 1975; Helfman et al. 1997). The vertical orientation of the body has affected the position of the fins. The paired pectoral and pelvic fins are in the typical location for fishes. However, the positions of the dorsal, caudal, and anal fins were modified (Fig. 3; Atz 1962; Klausewitz 1964). The posterior 25% of the vertebral column bends ventrally and displaces the median fins. The first dorsal fin is reduced to a spine that is situated longitudinally at the terminus of the body. The second dorsal fin, which is mobile, takes on a position more like a caudal fin, while the caudal fin is displaced ventrally close to the anal fin. Atz (1962) asserted that the positions of the fins were advantageous for the vertically oriented shrimpfish to move horizontally.

Shrimpfishes swim singly or in groups of up to 100 individuals in a head-down orientation so that the rounded dorsal edge of the body leads (Atz 1962; Klausewitz 1963, 1964; Helfman et al. 1997; Lieske and Myers 2005). Movements of the fins produce swimming actions. Beating of the pectoral fins and the second dorsal fin are considered responsible for propulsion, whereas the caudal fin and anal fin are used for steering (Klausewitz 1964; Consi et al. 2015). Groups of shrimpfishes can move in a synchronized manner changing direction rapidly by rotating about the longitudinal axis of the body (Atz 1962; Klausewitz 1964; Consi et al. 2015). Atz (1962) remarked that he and others found the shrimpfish to be highly agile and capable of rapid turns by rotating around the longitudinal axis while in a vertical orientation. However, no quantitative measurements were made to detail the swimming performance of shrimpfishes. It is interesting that closely related Macroramphosidae (snipefishes) has posteriorly placed median fins and a head-down orientation when stationary, but swims with the body oriented horizontally (de Oliveira et al. 1993).

The aim of this study was to examine the factors related to stability and maneuverability of the shrimpfish. By quantifying changes in the state of the fish with regard to the relative positions of the centers of mass and buoyancy, the unique body design and orientation can be assessed with respect to swimming performance. We hypothesized that the head-down orientation was the stable condition and therefore directly related to the position of the body and that maneuverability would be limited. To this end, we examined yawing (i.e., turning maneuvers), pitching, and maximum burst swimming speed for the shrimpfish.

## Materials and methods

Eight adult shrimpfishes (*Aeoliscus punctulatus*) were obtained for morphological analysis from the ichthyological collections of the National Museum of Natural History, Tel Aviv University, Israel (catalog number P5526), Smithsonian National Museum of Natural History (catalog number USNM84092), and the Philadelphia Academy of Natural Sciences (catalog numbers ANSP55069, ANSP55085, and ANSP97458). The preserved specimens had been stored in 70% ethanol, which may potentially cause some error in measurement. The specimens were measured from scaled photographs taken with a digital still camera (Sony Cyber-shot, model DSC-HX5V, 10.2 megapixels). The body length (BL) was measured from the tip of the rostrum to the base of the dorsal spine, the maximum thickness (*T*_max_) was the maximum transverse (i.e., side to side) distance, and the maximum body depth (BD_max_) was the maximum distance from the dorsal to ventral edges. For an indication of the streamlining of the body, fineness ratios (FRs) based on morphometric parameters were calculated as BL/*T*_max_ and *D*_max_/*T*_max_.

The position of the CM for the shrimpfish was determined based on the procedure of Alexander (1983). A shrimpfish was suspended by a string and photographed. The fish was then suspended by the string from another location on the body and photographed again. Using Adobe Photoshop, the two images were overlaid and the intersection of lines following the strings showed the position of the CM.

As opposed to the CM, which is determined by the weight in air and density of the body of the fish, the CB is based on the weight and density of the fluid displaced by the body. Based on the volume of displaced water, the position of the CB was determined geometrically, using the equation:

$$d=\frac{\sum M_{i}d_{i}}{M_{\mathrm{tot}}}$$

where *d* is the distance of the center of buoyancy from a fixed point, *M_i_* is the mass of an incremental segment of water corresponding to the position of a body segment *i*, *d_i_* is the distance from the center of the water segment to the fixed point and *M*_tot_ is the total mass of the water displaced. As the mass times the gravitational acceleration (*g*), is weight, *g* would cancel out from both the numerator and denominator of the equation leaving the position of CB based on the mass of water displaced. We estimated the mass of the water displaced for each section based on the height of each section, the foil-shaped cross section of the shrimpfish, digitized from a head-on picture, and the maximal body width. Sections were 1 mm each. Measurements were made from photographs of specimens, using the software ImageJ. The procedure was repeated for the longitudinal axis of the fish (proximal-distal) and its width (dorso-ventral axis) to determine the distance of the CB from the rostrum and the dorsal edge of the body. We repeated the measurements for *n* = 8 fish (Table 1). The longitudinal positions of the CM and the CB were indicated as a percent of BL from the rostrum and with respect to dorso-ventral axis as indicated as a percent of the local body depth (BD_local_) from the dorsal edge of the body.

**Table 1:** Morphometrics of shrimpfish (*A. punctulatus*)

Dimensions	Mean ± SD
Body mass (g)	4.6 **±** 1.6
Body length (L, cm)	13.7 **±** 1.4
Maximum thickness (*T*_max_, cm)	0.6 **±** 0.1
Maximum depth (*D*_max_, cm)	2.0 **±** 0.3
Rostrum to pectoral base (cm)	5.7 **±** 0.5
Rostrum to pelvic base (cm)	9.4 **±** 2.6
Rostrum to anal base (cm)	10.9 **±** 1.1
Rostrum to dorsal fin base (cm)	12.7 **±** 1.2
Length dorsal spine (cm)	0.7 **±** 0.1
Fineness ratio (*L*/*T*_max_)	24.9 **±** 2.7
Fineness ratio (*D*_max_/*T*_max_)	3.7 **±** 0.1
Rostrum to center of gravity (cm)	7.1 **±** 0.7
Center of mass to body length (%)	51.7 **±** 0.3
Center of mass to body depth (%)	40.4 **±** 1.6
Center of buoyancy to body length (%)	51.7 **±** 0.9
Center of buoyancy to body depth (%)	49.3 **±** 2.7

**Fig. 4:**
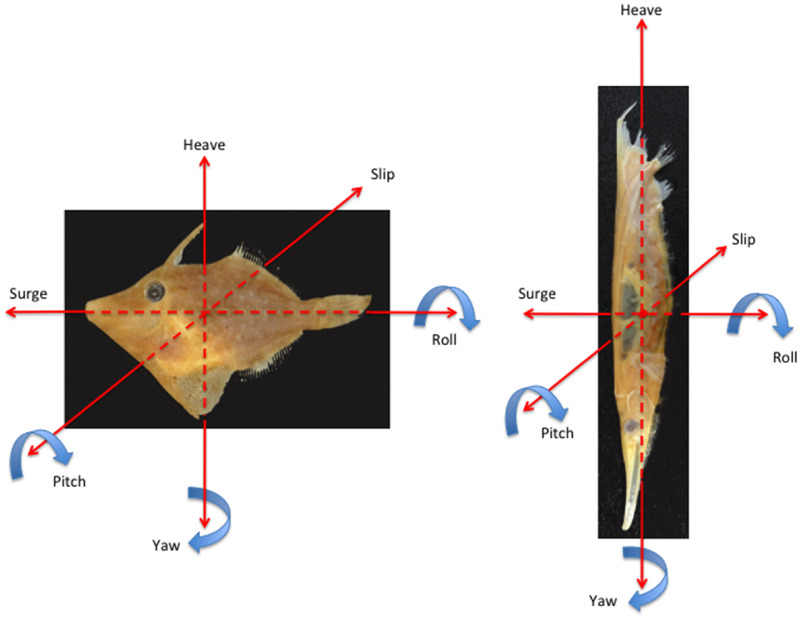
Degrees of freedom illustrated for a filefish in a typical horizontal orientation and a shrimpfish in a head-down orientation.

Examination of the swimming performance of shrimpfishes was performed at the Underwater Observatory Marine Park in Elat, Israel. Six shrimpfishes (*A.punctulatus*) were on display in a ∼60 L saltwater aquarium (∼50x30x40 cm WxDxH, respectively). The fishes were free to move about the aquarium and often oriented and moved in unison. The motions of the shrimpfishes are based on the six degrees of freedom on a free moving body in space. The degrees of freedom are relative to three orthogonal axes (horizontal, vertical, transverse). Linear movement along an axis is translational, where movement along the horizontal axis is surge, vertical axis is heave, and transverse axis is slip. Rotational movements about the three axes include roll about the horizontal axis, yaw about the vertical axis, and pitch about the transverse axis. In a typical fish (Fig. 4), the horizontal axis corresponds to the longitudinal axis and the vertical axis corresponds to the dorso-ventral orientation of the fish. However, for the shrimpfish in a head-down orientation (Fig. 4), the horizontal axis is associated with the dorso-ventral orientation for surge/roll and the vertical axis is associated with the longitudinal axis of the fish for heave/yaw. Yawing motions are turning rotations.

The movements of the shrimpfishes were video recorded with an SA3 Photron camera (San Diego, California) at 500 frames/s using Photron’s PFV software. The video recordings were analyzed with ImageJ (NIH; Schneider et al. 2012). Video records were sorted into three swimming behaviors: yawing, pitching, and burst swimming. The angular displacement during a yawing turn was determined by the fish executing a change of 90°. Pitching was defined as an upward angular displacement of the rostrum by rotation about the fish’s transverse axis from an initial head-down orientation. The horizontal plane was assigned an angle of 0° and the vertical plane was indicated as 90°. The angular position of the longitudinal axis of the shrimpfish relative to the horizontal plane was used to measure the change in pitch angle. Each of the rates of yawing and pitching was measured as the angular velocity in degrees/s. The average burst swimming speed was measured from the displacement divided by time that the fish moved horizontal while in the plane of the camera from a static position (i.e., speed = 0). As individual fish in the aquarium could not be measured, the average burst swimming speed was calculated as BL/s, based on the distance transited relative to the BL of the individual fish measured from the video. To assess the use of the dorsal, caudal, anal, pelvic, and pectoral fins during the three types of maneuvers, the movements of the fins were characterized as active (i.e., flapping) or passive (i.e., inactive). Comparisons among the fins were only made from video sequences in which all the fins could be observed.

To characterize the cross-sectional profiles along the body length of the shrimpfish, a specimen (ANSP55069) was examined. The specimen was 3D scanned with a GoMeasure3D scanner (CNC Services, Amherst, VA). The *stl file of the scan was created in MeshLab (v1.2.3) and the cross-sectional profiles were imaged at 25, 50, and 75% BL. The profiles were measured with ImageJ software (NIH, version 1.38). Measurements of the profiles at each location included the BD represented by the linear distance from leading to trailing edges (i.e., dorsum to venter of body), local thickness (*T*), and distance from the leading edge to maximum thickness (*S*). A thickness ratio (TR) of the sections (25, 50, and 75%) was computed as *T*/BD.

Our approach was to analyze the maneuvering performance of the shrimpfishes in an aquarium setting without manipulation. As performance for pitch, yaw, and maximum burst speed were not comparable, we limited our analysis of the maneuvering performance to descriptive statistics. To examine the maximal performance by the shrimpfishes for each variable, data were expressed as maximum values, means ± 1 standard deviation (SD), and the means ± SD of the highest 20% of values (i.e., maxima for yaw rate, pitch rate, burst velocity). The choice of the highest 20% of values was considered arbitrary but was used previously for comparisons of turning performance (Webb 1983; Gerstner 1999, Fish and Nicastro 2003, Fish et al. 2003). The use of fins was indicated as the percentage of maneuvers made by the shrimpfishes.

## Results

### Shrimpfish morphometrics

**Fig. 5:**
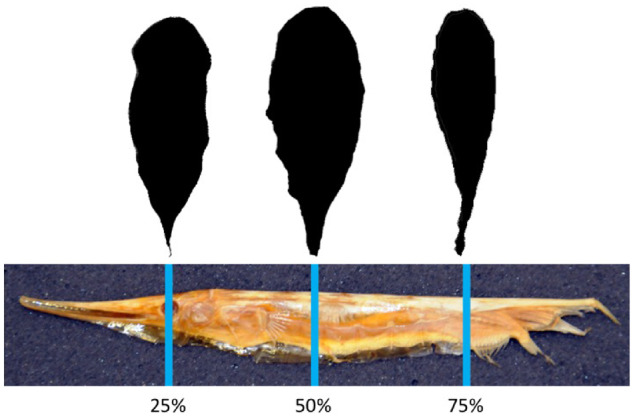
Cross-sectional profiles at 25, 50, and 75% of BL.

**Fig. 6:**
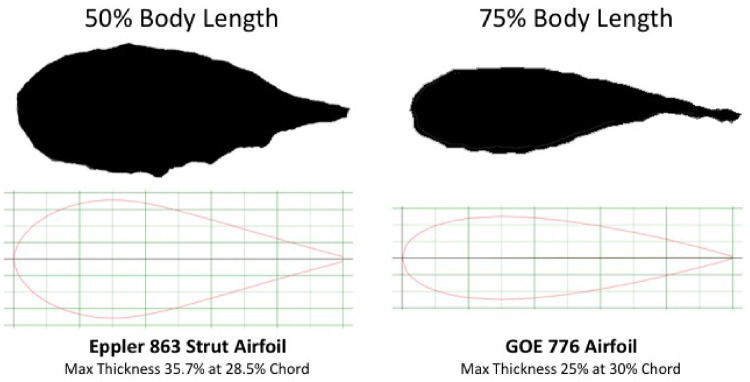
Cross-sectional profiles at 50 and 75% of BL in comparison with engineered foils from Airfoil Tools (2014; airfoiltools.com). Images of engineered foil outlines were provided with permission from Airfoil Tools.

The morphometrics of the eight shrimpfishes that were examined are provided in Table 1. FRs based on BL/*T*_max_ and *T*_max_/*D*_max_ were 24.9 ± 2.7 and 3.7 ± 0.0, respectively. FR based on BL/*T*_max_ was extremely high compared to other marine animals (Webb 1975; Fish 1993; Videler 1993) and about 6.7 times greater than FR based on *T*_max_/*D*_max_. The position of the CM was located at 0.52 ± 0.29 BL. The position of CB was 0.52 ± 0.93 BL. The position of CB was slightly ventral of CM (Fig. 2). The cross-sections of the body at 25, 50, and 75% BL approximated streamlined profiles with a fusiform design (Fig. 5). TR was 0.32, 0.38, and 0.26 at 25, 50, and 75% of BL, respectively, and the position of *S* was 0.41, 0.34, and 0.30 at 25, 50, and 75% of BL, respectively. The cross-sectional profiles at 50 and 75% were similar to the Eppler 863 Strut Airfoil and GOE 776 Airfoil, respectively (Fig. 6; Airfoil Tools, 2014; airfoiltools.com). Because the section at 25% BL was located at the eyes, the cross-section was not similar to published foil sections.

### Shrimpfish swimming

In the aquarium, shrimpfishes typically swam in a head-down orientation. The usual arrangement of the orthogonal axes (i.e., longitudinal, vertical, transverse) that is used to describe the rotational degrees of freedom (i.e., roll, pitch, yaw) in a 3D space are rotated 90° counterclockwise with respect to the longitudinal axis for the shrimpfish (Fig. 4). Yawing maneuvers about the longitudinal axis of the body performed directional changes (Supplementary Movie 1). A clockwise pitching could be performed to rotate the body to a nearly horizontal posture (Supplementary Movie 2). No significant rolling motions were observed that could tilt the body laterally. Burst swims were noted as rapid anterior movements of the dorsal aspect of the body. The six fishes normally swam together and often coordinated their movements.

**Fig. 7:**
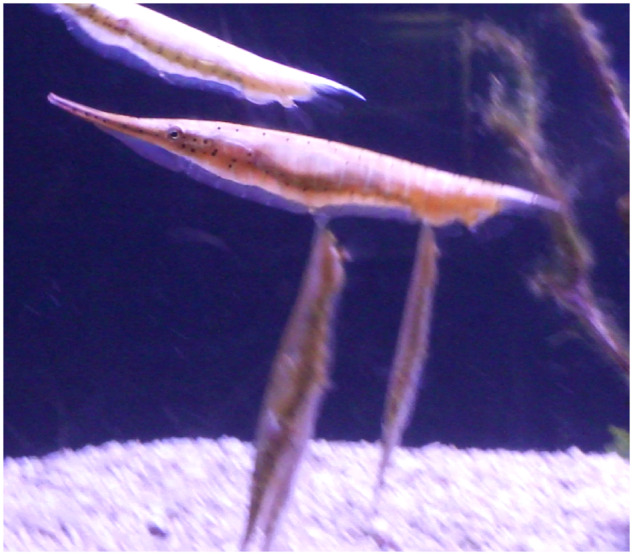
Shrimpfish in aquarium demonstrating the ability to pitch the rostrum dorsal above the horizontal.

A total of 119 video sequences were recorded of swimming for the shrimpfishes in the aquarium. These sequences were sorted into 62 for yawing (i.e., turning) maneuvers, 32 for pitching maneuvers, and 25 for maximum burst swimming speeds. Data on yawing, pitching, and burst swimming are summarized in Table 2. As measured from the high-speed video, the shrimpfishes were able to perform an upward pitching motion to a nearly horizontal orientation (0.3°). The mean angle at the end of a pitching maneuver was 19.8°±12.9° below the horizontal. Additional observations were made that showed that the shrimpfishes were capable of pitching their bodies by about 20° above horizontal (Fig. 7). The mean pitch rate was 59.0 ± 24.8°/s. The maximum pitch rate was 111.1°/s and the highest 20% of pitch rate was 99.2 ± 8.2°/s. The mean turn rate by yawing maneuvers was 257.2 ± 178.8°/s. The maximum turn rate was 957.4°/s and the highest 20% of turn rate of recorded maneuvers was 550.3 ± 168.4°/s.

**Table 2:** Summary of maneuvering performance by shrimpfishes

	Yaw rate	Pitch rate	Burst speed
	^o^/s	^o^/s	BL/s
*N*	62	32	25
Mean ± SD	257.2 ± 178.8	59.0 ± 24.8	1.0 ± 0.6
Maximum	957.4	111.1	2.3
Maximum 20%	550.3 ± 168.4	257.2 ± 178.8	2.0 ± 0.2

The shrimpfishes would hold station in the aquarium but could exhibit frenetic short bursts of speed either individually or in a group for short distances. The duration of burst swims ranged from 0.15 to 1.20 s. Mean burst swimming speed was 1.0 ± 0.6 BL/s. The maximum burst speed was 2.3 BL/s and the highest 20% of burst speed was 2.0 ± 0.2 BL/s. When accelerating, it was noted that the fishes displayed some upward pitching movements. In the vertical orientation when feeding, the shrimpfishes were observed to burst downward head first and then to ascend tail first using fin motions for propulsion.

The pectoral fins were constantly in motion, even when a shrimpfish was holding station. During these times the pectoral fins beat asymmetrically. Table 3 shows the proportion of video sequences that each fin was actively in use during each maneuver. The pectoral fins were used in all maneuvers, whereas the other fins showed varying amounts of use depending on the maneuver. The dorsal fin and anal fin were used 100% of the time when a shrimpfish executed yawing and pitching motions. The caudal fin was in use when pitching only 25% of the time during yaws. When not active during yawing maneuvers, the caudal fin was canted to one side to act like a rudder. Although the pectoral fin was always in use during burst swimming, the pelvic fin was never used, and the percentage of time that the dorsal fin, anal fin, and caudal fin were in use was 77, 69, and 31% of the time, respectively.

**Table 3:** Percentage (%) of video sequences in which fins were actively used during a maneuver

Fin	Dorsal	Caudal	Anal	Pelvic	Pectoral
Maneuver					
Yaw	100	0.25	100	58	100
Pitch	100	100	100	28	100
Burst	77	31	69	0	100

## Discussion

### Stability and orientation

The shrimpfish use both paddling and hydrostatic control to maintain its position and orientation. Paddling can be used to actively stabilize the body or create maneuvers. In particular, the continuous motions of the pectoral fins could resist perturbations due to changes in flow, which can occur suddenly. Such disturbances can transpire due to wave actions, currents, interactions with conspecifics, and predators. Alternatively, passive hydrostatic stability is dependent on a function of the moments created from the relative positions of the CM and the CB. The position of the CM for shrimpfishes was found to be the same as the CB along the longitudinal axis of the body. With the longitudinal arrangement of the CM and the CB, the head-down orientation would require the use of the beating fins to maintain stability for the shrimpfishes. This posture would be typical for the shrimpfish; however, the difference in positions of the CM and the CB along the depth axis could indicate that the stable orientation for swimming would be with a horizontal (=longitudinal) orientation as was claimed by Atz (1962).

Measurements of the CM and the CB on shrimpfishes made by Consi et al. (2015) indicated that the CM was slightly posterior and dorsal to the CB. These relative positions would create a moment that rotates the rostrum upward and makes the head-up posture the stable orientation. This result was contradicted by the relative positions of the CM and the CB reported in this communication and run counter to observations of shrimpfishes in the wild and in aquariums, where a head-up orientation was not described (Atz 1962; Klausewitz 1963, 1964; present study). The difference, particularly between the position of the CB by Consi et al. (2015) and the present study, may have been due to the dissimilar methods used. Consi et al. (2015) used an immersion method that was corrected for surface tension. The present study performed an analysis to determine the geometric center of the shrimpfishes and thus the geometric center of the displaced volume of water, which is the definition for the CB (Weihs 1993).

Stabilizing systems maintain a desired postural orientation and self-correct for both internal and external perturbations (Webb 1997, 2004b; Fish 2002). Highly stable systems rapidly react to disturbances and return a body to the original orientation and trajectory with minimal overshoot (Webb 1997). To control stability, restoring forces are developed to counter the disturbing forces that produce a destabilizing motion or change in motion (Webb 2000, 2004b). The restoring forces can be generated either actively or passively (Fish 2002; Webb 2006). Active mechanisms come with certain limitations. The active generation of the restoring forces can control the position and orientation of a body but for a cost of energy, and potentially produces uncontrolled motions that further destabilize the system. The destabilizing perturbation needs to be detected and analyzed before the appropriate restoring force can be generated (Webb 2000, 2004b). This response suffers a time delay that may reduce the effectiveness of precisely stabilizing the body in a dynamic system (Webb 2000; Cotel and Webb 2004). An attempted correction can potentially amplify the perturbation if the response period approaches half the period of the disturbance in what is referred to as “pilot-induced error” (Webb 2000). When subjected to a hydrodynamic disturbance, the creek chub (*Semotilus atromaculatus*) was found to be more stable with a response latency of 123 ms compared to smallmouth bass (*Micropterus dolomieu*) and bluegill sunfish (*Lepomis* macrochirus) with longer response latencies of ∼200 ms (Webb 2004a).

The use of passive stabilization mechanisms reduces the possibility of “pilot-induced error” and does not require the expenditure of additional energy. In addition, passive stabilization can function at low swimming speeds. Active stabilization uses the flow in concert with lift-producing control surfaces to generate the forces to counter perturbations (Webb 2000). As lift is a function of the velocity squared, low flows generate less hydrodynamic force to stabilize the body against large perturbations than high flows (Marchaj 1988; Webb 1997). In low-flow systems, stability can be controlled by active paddling and/or hydrostatic mechanisms (Vogel 1994). Hydrostatic mechanisms arise from density differences (Webb 2006). Hydrostatic stability uses buoyancy to generate the forces and moments on a submerged, stationary body to return it to a stable orientation after it was disturbed (Weihs 2002). In many fishes, the position of the CB is largely due to a low-density gas-filled inclusion, the swim, or gas bladder (Breder 1926). Many fishes are considered to be hydrostatically unstable, whereby the CM is typically above the CB making it unstable in roll (Webb and Weihs 1994; Eidietis et al. 2002; Cotel and Webb 2004). Due to the imbalance from the relative positions of the CM and the CB, the movement of the control surfaces (i.e., fins) is necessary to balance the destabilizing hydrostatic moments. However, it was argued that CM and the CB coincide allowing fishes such as labrids and scarids to sometimes swim on their sides (Breder 1926). Webb and Weihs (1994) showed that slow swimming and station-holding fishes (e.g., bluegill sunfish) have the CM slightly anterior to the CB but at the same level along the longitudinal axis of the body.

### Pitching and turning maneuvers

As opposed to stability, maneuverability is a state in which instability occurs and is controlled. A maneuvering body undergoes translation or rotation as opposed to a stable body in which the sum of all forces and all turning moments are zero (Webb 1997; Fish 2002). Morphological design that enhances stability, constrains maneuverability (Weihs 1993, 2002; Webb 2000; Fish 2002; Fish et al. 2003). The terms “stability” and “maneuverability” are in effect antonyms and mirror images of each other (Weihs 1993; Webb 1997). The close proximity of the CM and the CB in shrimpfishes would enhance maneuvering capabilities. However, the structure, fin position, and body orientation of the shrimpfishes indicate a stable morphology that would hinder rapid maneuverability with respect to yaw, pitch, and roll. Indeed, yaw and roll would be limited by the long, stiff, and highly compressed body of the shrimpfishes that resists turning and lateral tilting. In addition, the relative positions of the CM and the CB with the head-down posture generate a pendulum-like stabilizing moment.

Despite the hydrostatic stability exhibited by the shrimpfishes, pitching motions were possible using the actions of the various fins (pectoral, dorsal, caudal, and anal). The shrimpfishes can rapidly pitch the body into a more horizontal posture. However, for this maneuver, Klausewitz (1963, 1964) indicated that the shrimpfish could not attain a horizontal orientation and could only rotate its body upward 20° from the vertical.

Klausewitz (1963, 1964) and Atz (1962) stated that the shrimpfish could swim rapidly and was highly maneuverable. The fish was reported to turn about its longitudinal axis quickly and precisely (Atz 1962). Indeed, Consi et al. (2015) considered that the shrimpfish could serve as a model for a highly maneuverable autonomous underwater vehicle (i.e., biomimetic robot).

**Fig. 8:**
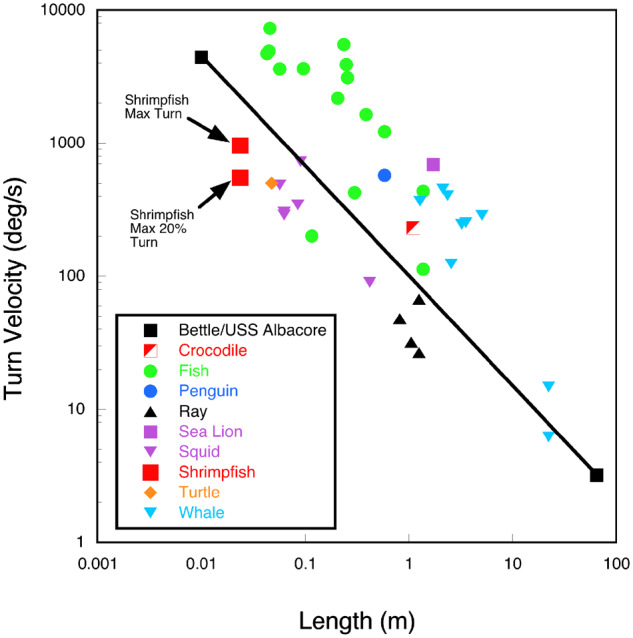
Comparison of turning rates of shrimpfish with other aquatic animals. Symbols above the solid line represent animals with flexible bodies and symbols below the line represent animals with rigid bodies. Data are from Webb (1976, 1983), Hui (1985), Foyle and O’dor (1988), Miller (1991), Blake et al. (1995), Gerstner (1999), Walker (2000), Frey and Salisbury (2001), Fish (1997, 2002), Fish and Nicastro (2003), Fish et al. (2003, 2018), Kajiura et al. (2003), Domenici et al. (2004), Rivera et al. (2006), Parson et al. (2011), Jastrebsky et al. (2016, 2017), Helmer et al. (2016), Geurten et al. (2017), and Segre et al. (2018).

Turning is performed by a yawing maneuver by rotation about the longitudinal axis of the shrimpfish. The torque necessary for this maneuver was initiated by active use of the pectoral, dorsal, and anal fins with the caudal fin used as a passive rudder. Rotation about the longitudinal axis has a low moment of inertia. Therefore, the maximum angular change by yawing is 8.6 times faster than the maximum pitching rate. The ability to change direction by yawing can exceed the turning performance of other aquatic animals with rigid bodies of similar body length (Fig. 8). Rigid-bodied fishes such as the boxfish (*Ostracion meleagris*) and cownose ray (*Rhinoptera bonasus*) turn at 147.0 and 48.0°/s, respectively (Walker 2000; Parson et al. 2011). Squid (*Lolliguncula brevis*) and cuttlefish (*Sepiabandensis*) have a maximum turn rate of 303.6 and 485.0°/s, respectively (Jastrebsky et al. 2016, 2017). These animals have a longitudinal axis parallel to the horizontal plane and turn by yawing. Therefore, the rigid-bodied fishes and cephalopods have moments of inertia greater than the shrimpfish. If the shrimpfish was modeled as a solid cylinder rotating about its longitudinal axis, the moment of inertia is calculated as 0.5 MR^2^ to equal 4.5 × 10^−7 ^kg m^2^, where *M* is the body mass (kg) and *R* is the radius (m) (Freedman et al. 2018). When modeled as a solid cylinder rotating about the center of its length (BL, m), the moment of inertia is calculated as 0.25 MR^2^ + 0.0833 MBL^2^ and is equal to 1.1 × 10^−5 ^kg m^2^. The moment of inertia for a shrimpfish turning around the center of its body length (yawing) would be 25 times greater than the fish performing a roll. A turn performed by rolling would be more likely to reorient the body faster than by yawing.

Other animals can roll rapidly. Birds and sea lions (*Zalophus californianus*) will quickly bank to change direction (Norberg 1990; Fish et al. 2003). Using a zero angular momentum turn, alligators (*Alligator mississippiensis*) perform the “Death Roll” at rates ranging from 257 to 978°/s to dismember prey (Fish et al. 2007). Spinner dolphins (*Stenella longirostris*) start spinning underwater before breaching the surface of the water (Fish et al. 2006). Once the water is cleared, the spin rate of the dolphin increases due to conservation of angular momentum. The dolphins are able to execute up to seven complete revolutions before splashing down in the water to dislodge remoras (Fish et al. 2006; Weihs et al. 2007). Even the largest animal on the planet, the blue whale (*Balaenoptera musculus*), rolls. The whale will roll at rates up to 48°/s when foraging in order to localize patches of krill (Goldbogen et al. 2012; Segre et al. 2016, 2018).

### Burst swimming

The cross-sectional streamlined body of a shrimpfish allows it to perform burst swims. Atz (1962) characterized its rapid swimming as “cleaving the water with its razor-shaped body” (Atz 1962; Norman and Greenwood 1975; Consi et al. 2015). The optimal FR to minimize the drag on a streamlined body is 4.5–7 (Gertler 1950; Webb 1975; Blake 1983). If a shrimpfish swam longitudinally as a typical fish, FR (= BL/*T*_max_) would be 300% greater than the maximum optimal value, whereas in its typical swimming posture the FR (= *D*_max_/*T*_max_) is only 20% lower than the minimum optimal value. The fusiform profile of shrimpfishes emulating engineered profiles would aid in minimizing drag during burst swims.

Webb (1975) defined burst swimming as a high activity maintained for <15 s. The rapid motions of the shrimpfishes fell under this definition. Compared to other fishes, the maximum velocity of the shrimpfishes was low. Domenici and Blake (1997) compiled a listing of the maximum velocities of fishes, which ranged from 3.3 to 27.3 BL/s. In all cases, flexible-bodied fishes bent their bodies in a classic C- or S-start to accelerate (Webb 1976; Webb and Skadsen 1980; Domenici and Blake 1991, 1997). The rigid body of the shrimpfishes prevents them from displaying high speeds within the range reported by Domenici and Blake (1997). As dimension *D*_max_ is the characteristic length in the direction of travel and flow, it can be exchanged for BL to calculate the relative burst swimming speed for the shrimpfishes. Based on the measurements of the shrimpfishes in Table 1, the average and maximum speeds were 6.2 and 14.1 *D*_max_/s, respectively. In this case, the maximum swimming speed for the shrimpfishes is within the range of maximum speeds for flexible-bodied fishes.

### Summary

Shrimpfishes evolved to swim and maneuver in a head-down posture. The CM was positioned in close proximity to the CB. This arrangement of the two centers would permit enhanced maneuverability in the head-down orientation, but stability would be dependent on the use of the fins for shrimpfishes. The cross-sectional profile of the body is streamlined with a fusiform design. Despite the rigid body, the shrimpfish is able to use its mobile fins to pitch the body upward into a horizontal orientation, turn and change direction using a yawing maneuver about its longitudinal axis, and accomplish relatively high-speed swimming.

## Supplementary Material

obz025_Supplementary_Data
